# Effects of Different Long-Term Exercise Modalities on Tissue Stiffness

**DOI:** 10.1186/s40798-022-00462-7

**Published:** 2022-06-03

**Authors:** Ewan Thomas, Salvatore Ficarra, Masatoshi Nakamura, Antonio Paoli, Marianna Bellafiore, Antonio Palma, Antonino Bianco

**Affiliations:** 1 Sport and Exercise Sciences Research Unit, Department of Psychology, Educational Science and Human Movement, University of Palermo, Via Giovanni Pascoli 6, 90144 Palermo, Italy; 2 Faculty of Rehabilitation Sciences, Nishi Kyushu University, 4490-9 Ozaki, Kanzaki, Saga 842-8585 Japan; 3 Department of Biomedical Sciences, University of Padova, Padua, Italy

**Keywords:** Stiffness, Stretching, Resistance training, Aerobic, Plyometric exercise

## Abstract

Stiffness is a fundamental property of living tissues, which may be modified by pathologies or traumatic events but also by nutritional, pharmacological and exercise interventions. This review aimed to understand if specific forms of exercise are able to determine specific forms of tissue stiffness adaptations. A literature search was performed on PubMed, Scopus and Web of Science databases to identify manuscripts addressing adaptations of tissue stiffness as a consequence of long-term exercise. Muscular, connective, peripheral nerve and arterial stiffness were considered for the purpose of this review. Resistance training, aerobic training, plyometric training and stretching were retrieved as exercise modalities responsible for tissue stiffness adaptations. Differences were observed related to each specific modality. When exercise was applied to pathological cohorts (i.e. tendinopathy or hypertension), stiffness changed towards a physiological condition. Exercise interventions are able to determine tissue stiffness adaptations. These should be considered for specific exercise prescriptions. Future studies should concentrate on identifying the effects of exercise on the stiffness of specific tissues in a broader spectrum of pathological populations, in which a tendency for increased stiffness is observed.

## Key Points

• Resistance training increases tendon stiffness in healthy subjects, but limited evidence is available in pathological populations.
• Low-load resistance training, aerobic training and stretching promote arterial stiffness reduction.
• It is still premature to infer that exercise may chronically modulate peripheral nerve stiffness; however, mechanical elongation seemingly has some effects.

## Introduction

Stiffness is a mechanical property of tissues, defined as the ability to oppose deformation when mechanically stressed [1, 2]. Different factors need to be taken into account, such as stress, which is the stimulus applied to the tissue and strain, which represents the deformation caused by the stress. Therefore, stiffer tissues will deform less when compared to softer tissues [3]. Another important concept of biological tissues is that these are viscoelastic, both possessing solid and liquid components [4]. Hence, due to their viscous components, tissues will deform less if stress is applied quickly [4]. Such concepts help us to understand that tissues rich in water that are also less dense, have lower stiffness and may deform more easily, meaning that they will be able to accommodate movement if required and that different stressors will result in different effects. Different tissues based on their biological function express different intrinsic stiffness levels. An example may be seen in fat, which is usually soft, with one of its functions being to cushion vital organs and bone which is rigid with the function to withstand important mechanical loads.

Tissue stiffness has been recently discovered to possess a role in orienting cell division, maintaining tissue boundaries, directing cell migration or driving differentiation [5, 6]. This means that variations in tissue stiffness may lead to altered physiological states, leading to pathological development [7]. The interaction between cells and the extracellular matrix drives tissue adaptations and factors such as non-uniform compressions of the extracellular matrix, internal cellular physical constraints (cell tractions or increased density) and cell jamming or the propagation of compressive forces along a tissue are the main causes of increased tissue stiffness mainly by fibroblast activation [8, 9]. Trauma may lead to formations of scars [10] which may impair mobility; altered biomechanical loads may lead to calcific formations [11] causing pain and reducing independence; ageing or lifestyle modifications can lead to atherosclerosis [12], an important independent factor increasing cardiovascular risk, or incorrect eating habits or hereditary factors may cause liver cirrhosis [13] a life-threatening disease. All are conditions which have in common two key points, altered tissue stiffness due to increased fibrosis [2] and reduced quality of life. However, it is also important to highlight that an adequate level of stiffness achieved by external mechanical forces is fundamental in order to maintain homeostasis. For example, remodelling of bone and cartilage needs external loading for calcium deposition or proteoglycan stimulation to avoid bone fragility and cartilage dysregulation, which are frequently observed in no-gravity environments or as a consequence of disuse [5].

Since strain and stress may vary considerably in vivo, measuring tissue stiffness is particularly challenging. To date different methods are employed to measure tissue stiffness, ranging from myometers which are mainly employed for musculoskeletal purposes for evaluating myofascial stiffness, a condition frequently associated with pain [14], to ultrasound elastography (which can be applied in two main methods (1) strain imaging that uses internal or external compression stimuli and (2) shear wave (SW) imaging that uses ultrasound-generated travelling shear wave stimuli) as a non-invasive technique for the assessment of organs such as liver, kidneys, thyroid, prostate or lymph nodes [15] but also for other soft tissues such as nerves, fascia, tendons, ligaments and muscles [16].

All the above-mentioned disorders and the methods developed to measure stiffness imply a modification from a physiological to a pathological condition. Nevertheless, there are different therapeutic strategies employed to reduce tissue dysfunction and reverse stiffness adaptations. These may involve both pharmacological and non-pharmacological interventions, for example, beta-blockers or ACE inhibitors [17] and nutritional therapy [18] for increased arterial stiffness, manual therapy for myofascial stiffness [19] or exercise for different conditions [20–23].

Another important implication regarding tissue stiffness is shear displacement of connective tissue. This latter wraps muscle fibres and muscle fascicles and during muscle contraction the rate of force development changes in relation to geometrical modifications of the connective tissue [24]. It has been proven that intramuscular connective tissues not only transmit passive forces through muscle fibres [25] but also allow the transmission of active generated forces which are not transmitted along tendons [26]. Interestingly, stiffness of muscles has been observed to change if the skin covering that muscle is removed, which in turn also modifies the rate of force development of that specific muscle [27]. Therefore, tissue stiffness modification may also have a role in sports performance due to its role in force transmission.

Despite exercise interventions being currently used to effectively treat pathological conditions or to improve health outcomes, there is still a lack of knowledge about the physiological adaptations induced by different exercise regimes for specific tissue stiffness modifications and therefore which should be the appropriate exercise modalities adopted. Therefore, the current review aims to clarify aspects related to changes in tissue stiffness as a consequence of long-term physical exercise. In addition, different forms of exercise will be taken into account to identify specific exercise-induced tissue stiffness adaptations. Combined forms of exercises or acute protocols will not be discussed since these study designs would not allow the defining of a specific form of tissue stiffness adaptation.

## Methods

This article was conducted to narratively review studies that addressed changes in tissue stiffness as a consequence of long-term physical exercise. To retrieve eligible articles, a manual search was conducted on the following database: PubMed, Scopus and Web of Science. The search was conducted between the 1st of September 2021 and the 31st of October 2021. Articles published up to the 1st of September 2021 were included, with no limitation on the beginning of the search period. The following search strategy was adopted to identify eligible articles: (stiffness) AND ((aerobic) OR (resistance training) OR (stretching)) AND ((muscul*) OR (vascular) OR (nerv*) OR (tendon) OR (fascia) OR (connective)). This search was extended utilizing the bibliography within the recruited texts. Articles published in any language were considered. To summarize the findings of this review, we divided the review into subsections, each of which elucidated adaptations to a specific form of exercise (resistance training, aerobic training, plyometric training and stretching). Main outcomes are reported in Table 1, and a classification of the strength of evidence for each outcome is provided. These are based on the recommendations provided by the Grades of Recommendation Assessment, Development and Evaluation (GRADE) Working Group and the Agency for Healthcare Research and Quality (AHRQ) [28].

## Results

The effects of different exercise modalities have been classified according to the different targeted tissues, which are classified as muscular, tendinous (for the connective tissue), blood vessels and peripheral nerves. A synthesis of the studies can be found in Table 1.

**Table 1:** Synthesis of the effects for each exercise mode on tissue stiffness or associated pathological condition

	Resistance training	Plyometric training	Aerobic	Stretching
*Tissue*				
Muscle	↕^c^	↑^c^	↕^c^	↓^cPF^ ↕^cO^
MTU	↕^c^	/	/	↓^cP^
Tendons	↑^a^	↕^c^	/	↕^c^
Vessels	↓^bLL^ ↑^bHL^	/	↓^a^	↓^b^
Nerves	/	/	/	↓^c^
*Pathology*				
Tendinopathy	↕^c^	/	/	/
Hypertension	↓^c^	/	↓^c^	↕^c^

Strength of evidence: a: High; b: Moderate; c: Low or insufficient

↑: Increase; ↓: decrease; ↕: unclear; /: no evidence; MTU: muscle tendon unit; HL: high load; LL: low load; P: passive stretching; PF: plantar flexors; and O: other muscles

### Muscular Tissue

Studies on muscle stiffness presented large variability in terms of protocols and assessment modalities. In addition, some studies also evaluated the effects of muscle tendon unit (MTU) properties. These differ in paradigms, since if MTU stiffness properties are assessed through ultrasonography, these may reflect aspects related to the muscular tissue [29], while evaluation through passive resistive torque at a given range of movement of a joint mainly reflects tendon properties [30], and therefore, these will be discussed separately in the muscle tendon unit section below.

#### Resistance Training

Despite consistent evidence regarding muscle adaptations to exercise being to date available, information pertaining to muscle stiffness after longitudinal resistance training (RT) protocols is scarce. A general overview highlights conflicting information, which also differs regarding exercise paradigms (i.e. forms of contraction, protocols and participants).

Within the single analysis of the retrieved protocols, two studies have found no significant effects on different targeted muscles, using ultrasound SW elastography (SWE) [31, 32]. The study by Akagi et al. found no effects of time or group on shear modulus, after the implementation of a six week, three days a week RT for the triceps brachii in sedentary and active participants [31]. The protocol consisted of 5 sets of 8 repetitions at 80% of 1RM (1 repetition maximum) of triceps brachii extensions, using dumbbells. The second study applied to a population of sedentary individuals, two RT programs with equated volume (at 67% of 1-RM), but with different weekly frequencies (1 session/week or 3 sessions/week – 6 sets of 12 repetitions or 2 sets of 12 repetitions, respectively) [32]. Despite differences in weekly frequency, no difference in muscle stiffness of the quadriceps was noted in either of the groups.

An eccentric hamstring training was tested by Uysal et al. in physically inactive individuals [33]. The authors compared eccentric hamstring training to concentric leg curl training relative to quadriceps and hamstring muscles characteristics. Progression was designed according to both weekly frequency and volume. Sets and reps increased from 2 × 5 to 3 × 12 for the eccentric training and from 1 × 10 to 3 × 8–12 for the leg curl training, during the 8 weeks of training. The administered exercises were carefully supervised to focus on eccentric or concentric contractions only. After the 8 weeks of eccentric training, participants’ thigh muscle stiffness decreased, while it was increased after the concentric protocol in both the antagonist’s muscles. In conclusion, this work suggests that a relationship between different contraction typologies and muscle stiffness could exist. However, compared to the above-mentioned studies, a myotonometer was adopted to measure muscle stiffness instead of elastography.

A different exercise regime was administered by Santos et al. in order to assess maximal concentric or eccentric training effects on vastus lateralis muscle characteristics [34]. Sedentary participants who were recruited for 15 weeks had to perform, at different range of motion (ROM) [35] angles, a leg extension exercise. Quasi-static elastography was used to evaluate participants. In contrast to the previous study, increased stiffness was noted regardless of contraction mode. Lastly, another study tested the effects of a squat and leg extension RT, on 15 inactive healthy men. The 8-week progressive intervention comprised 4 sets for each exercise during the first four weeks, and 6 sets between the fifth and the last training week. Weight was progressively increased to maintain repetitions in a 8–12 range. The participants were instructed to perform concentric contractions until exhaustion. At the end of the intervention, these showed an augmented vastus lateralis shear modulus (increased stiffness) [36].

Due to the contradictory results, the effects of RT on muscle are still unclear. Common points of all studies are that the relative intensity was greater or equal to 65%RM, while differences regarding contraction mode, volume, training frequency, duration of intervention and stiffness assessment techniques were present.

#### Plyometric Training

Limited evidence exists regarding muscle stiffness adaptations to plyometric training [37–41]. The retrieved studies performed either 14 [37], 12 [38], 8 [39], 6 [40] or 4 [41] weeks of training and evaluated the stiffness of muscles pertaining to the anterior and posterior compartment of both thigh and leg [40] and the plantar flexor muscles [37–39, 41]. Different exercise regimes were carried out; however, all involved jumps, either horizontally [40], vertically from heights which varied from 20 [38] to 80 cm [39] or both [41]. Two investigations were carried out by Fouré et al. [37, 39], which determined passive stiffness of the gastrocnemius muscle through B-mode ultrasonography. In both studies, an increase in muscle stiffness was observed. In particular, the 2009 study [39] reports a 33% increase after the intervention period. The study of Kubo et al. [38] differentiated between active and passive stiffness. The results of such investigation highlight that no difference in the passive components was present, but a significant increase was observed in the active components. The study of Mroczek et al. [40] measured stiffness through a handheld myometer (Myoton PRO) at the end of each week, for 6 weeks, noticing that an increase was present after each evaluation. Such increases were more pronounced in the tibialis anterior and the quadriceps muscles. Lloyd et al. [41] evaluated school children of 9, 12 and 15 years of age. Stiffness was indirectly measured through mathematical interpolation of body mass and performance parameters previously determined during functional assessment (jump height and ground contact time) at the beginning and after four weeks of training. It was noted that no increase was present in the 9-year-old group, while an increase was observed in either the 12- or the 15-year-old age groups. Such increase was observed for absolute and relative (normalized to body mass) muscle stiffness of the plantar flexor muscles.

There is concordance between studies despite different exercise regimes that plyometric training can increase the stiffness of the targeted muscles. However, further investigations are needed to confirm such findings.

#### Aerobic Training

Only one study assessed the effect on muscle stiffness after a specific form of aerobic training (AT). This work was carried out during a mountain ultra-marathon (330 km) competition in Italy [42]. Participants’ quadriceps muscle stiffness was analysed through SWE. Muscle stiffness resulted lower at the end of the race. The lower shear modulus was probably due to muscular inflammation and swelling caused by the prolonged extreme muscle recruitment. However, it is not possible to determine if changes in stiffness were caused by aerobic activity, running activity, prolonged muscular exertion, the slope differences during the race or a combination of factors. Only a partial return to baseline levels was observed after recovery (> 48 h post-race) [42].

The lack of additional records regarding AT on muscle stiffness suggests that more studies with a longitudinal design are required to underline adequate conclusions about muscle stiffness responses.

#### Stretching

Many investigations evaluated changes in muscle stiffness after acute bouts of stretching. The overall results of these studies indicate that a reduction in stiffness is present following the stretching interventions, especially if performed at high intensity [43]; however, such decreases subside within 10–20 min from the applied exercise [44].

However, few longitudinal interventions are present addressing outcomes related to stretching and stiffness. All the retrieved studies applied passive stretching interventions [45–52], except for that of Halbertsma and Göeken who applied a contract–relax (PNF) protocol [53]. The interventions had a length of 3 [48], 4 [45, 46, 52, 53], 5 [51], 6 [49] and 12 weeks [47, 50]. Stiffness was assessed in five studies through passive resistance to a stretch [45–47, 49, 53] and in three studies using SWE [50–52]. Only Blazevich et al. [48] used B-mode ultrasonography.

In detail, Blazevich et al. [48] applied a 30 s stretch to the plantar flexor muscles twice a day. A decrease in passive muscle stiffness at the end of the intervention, despite no change in tendon stiffness, was observed. Similar results are also reported by Akagi and Takahashi [51], who applied a six times a week, 3 × 120 s protocol to the gastrocnemius muscles. Also Andrade et al. [50] targeted the gastrocnemius muscles and the hamstrings, however only results pertaining to the gastrocnemius muscle were reported. The authors applied either muscle-directed stretching or nerve-directed stretching. The nerve-directed stretching showed no effect on the stiffness of the muscles, resulting, however, in decreased nerve stiffness. The muscle-directed stretching resulted in a significant decrease in the targeted muscles, without a reduction of nerve stiffness.

From the analysed manuscripts, all stretching protocols pertaining to the plantar flexor muscles elicited a decrease in the levels of evaluated stiffness.

The interventions of Marshall et al. [45] (5 times a week, 30 s stretch performed for 3 sets), Ichihashi et al. [52] (3 times a week, 5-min stretch), LaRoche and Connolly [46] (3 times a week, 30 s stretch performed for 10 sets) and Reid et al. [49] (5 times a week, 30 s stretch performed for 3 sets) applied stretching to the hamstring muscles. No univocal outcome was retrieved since a decrease [45, 52], no change [46] and an increase [49] in muscle stiffness were observed. It is important to underline that the only intervention which specified the intensity of the stretch was that of Marshall et al. [45], in which a maximal tolerable was used. In addition, also the study of Beltrão et al., which had the longest protocol, did not show any significant variation in either muscle or joint stiffness of the hamstrings and knee, respectively, at the end of the intervention [47]. These findings suggest that neither length nor stretching typology may specifically determine stiffness adaptations of the hamstring muscles. Similarly, the study of Halbertsma and Göeken [53] did not appraise a change in the hamstring stiffness following the stretch intervention. This last result may have been further influenced by the specific type of stretching (PNF) which mainly acts on adaptations of the neural circuitry [54]. Interestingly, two systematic reviews have evaluated acute [55] and long-term [56] effects of stretching regarding the range of motion, and these show that PNF provides the greatest increase after acute stretching, while static modalities are superior for long-term adaptations. Since four main mechanisms (sarcomerogenesis, stiffness, neural circuitry and reduced pain sensitivity [56]) are responsible for range of movement increases, and one of these is a modification of the stiffness of the tissues, it is plausible that range of movement increases related to PNF stretching may not primarily act on such parameter in the muscular tissue.

### Tendinous Tissues

All the reviewed literature focused on tendon stiffness. No studies concerning ligamentous or fascial stiffness following exercise were retrieved.

#### Resistance Training

A review conducted by Buchanan and Marsh in 2002 exploring the biomechanical and structural properties of tendons following exercise highlighted that, at the time, limited knowledge was present regarding such a specific topic. However, despite inconsistencies across studies a general increase in tendon stiffness was observed following RT. Recent evidence confirms that the loading of a tendon increases its stiffness by adapting through a modification of its elastic properties rather than morphological modifications (increased cross-sectional area) in an intensity driven direction [57]. Such modifications do not seem to depend on the type of muscle contraction. A study conducting RT in the plantar flexors for 5 days a week at 70% of 1RM observed an increase in stiffness of 18.8 ± 10.4% with no modification in hysteresis. Therefore, strength training increased tendon stiffness without modifications in the viscosity of the tissue [58]. A similar outcome was observed in another study which applied 80% of 1RM for 12 weeks, four times per week with an increase of 55 and 30% after static and dynamic training, respectively [59]. Morrissey et al. [60] performed six weeks of concentric and eccentric RT on the calf muscle twice a day every day applying 3 sets of 15 repetitions using participants’ body weight as functional overload. No difference was observed following concentric training, while a decrease was observed after eccentric training. Similar results were also retrieved by Ishigaki and Kubo following a similar protocol of 15 repetitions three times per week for twelve weeks, in which no differences were observed in tendon stiffness compared to baseline measures [61]. Interestingly, another work from Kubo et al. [62] tested the effects on tendon stiffness of an isometric exercise protocol. Participants had to complete four sessions/week for three months maintaining 15 s of isometric contraction during knee extension exercise. The isometric contraction was repeated 10 times, resting 30 s between contractions. Although tendon stiffness returned to baseline after 2 months of follow-up (5 months from the beginning of the intervention), a significant increase (54%) was observed at the end of the intervention. Similar morphological adaptations are also seen when comparing low-load blood flow restriction RT to high-load RT programs [63]. A 14-week training was carried out consisting of either a blood flow restriction training performed with a load of 20%1RM progressively increased to 35%1RM and a high-load training program ranging from 70 to 85%1RM. At training cessation, tendon stiffness, measured through ultrasound, resulted in a 36.1 and a 40.7% increase, respectively, compared to baseline evaluation.

Increased tendon stiffness seems also linked to the duration of the interventions [57]. However, Massey et al. compared the stiffness of the patellar tendon in men performing heavy RT, who had been training for one and four years to untrained controls. Tendon stiffness was significantly greater in the exercise groups, but no differences were observed between the one- and 4-year training experience groups [64]. Very similar results were observed in a group of old adults who underwent 1.5 years of RT. Increases in stiffness were observed up to 14 weeks with no changes in the subsequent evaluation [65]. These results highlight that adaptability is present in both young and older adults, and that such adaptability does not increase linearly, early reaching a plateau. All measures above described have been derived through a combination of B-mode ultrasonography and dynamometry.

When comparing measures of tendon stiffness across populations, it seems that independently of the type of sport, tendon stiffness is increased in athletes compared to controls [66, 67]. Increased tendon stiffness is also observed in older adults practising RT [68, 69]; despite this, it is still not clear if, in such a population, differences may occur following eccentric or concentric contractions [70].

Outcomes relating to RT and tendon pathology provide different results. Studies evaluating patellar tendinopathy found a general decrease in tendon stiffness following RT programs, performing either heavy loads or eccentric exercise [71, 72]. The heavy-load RT training consisted of a progressive loading program over 12 weeks which started by performing 4 sets of 15 repetitions each of three bilateral exercises (squat, leg press and hack squat) and ended with 4 sets of 6 repetitions each (weight was increased every two weeks) of the same exercises, while the eccentric exercise program involved standing on the affected leg on a 25° decline board and maintaining an upright trunk while slowly squatting down to the point of pain. This exercise was carried out twice a day for 12 weeks, performing 3 sets of 15 repetitions each. Conversely in Gatz et al. [35], who evaluated Achilles tendinopathy, stiffness increased following either an eccentric or an eccentric plus isometric protocol. The protocols consisted of either performing 3 sets of 15 repetitions twice a day for three months of eccentric exercises (lowering the body from a tiptoe position) or performing the same protocol including an extra day of isometric contraction (5sets of 45 s each) during the tiptoe position. The study of Gatz et al. also highlights that when comparing the symptomatic to the asymptomatic tendons, these possessed a lower stiffness. Results overall indicate that exercise interventions in pathological populations tend to induce a return to physiological stiffness values. To the best of our knowledge, no other study evaluated the long-term effects of RT on the stiffness of the tendons in patients suffering from tendinopathy.

Only one study took into account the possible relation between tendon stiffness in runners and parameters regarding oxygen consumption, following RT [73]. The study conducted a 14 week RT in runners, comparing stiffness of the triceps surae, the tendon aponeurosis and oxygen consumption to a control group. The resistance exercise group increased tendon stiffness by approximately 16%, and this also allowed a reduction of approximately 4% in oxygen consumption. These results indicate that in runners an increase in tendon stiffness may increase running economy.

The retrieved studies regarding tendon stiffness indicate that RT increases stiffness in healthy subjects. Despite limited evidence available in pathological populations, exercise interventions have the potential to induce a stiffness adaptation towards a physiological condition.

#### Plyometric Training

Few studies evaluated the effects of plyometric training on tendon stiffness [37–39, 74, 75]. No significant changes were observed in passive tendon stiffness except for Fouré et al. [75]. All studies, however, observe an incremental trend. Only one study observed a significant increase in active but not passive tendon stiffness [38]. All protocols differed significantly in terms of drop jump heights which ranged from 20 cm [38] to 80 cm [37].

It is to be noted that the majority of studies also taking into account muscular aspects highlight an increase in muscle stiffness following this form of exercise, which may explain the incremental trend observed. Muscular aspects have been discussed in the Muscular Tissue section above.

#### Stretching

Similarly to adaptations of the muscular tissue, many stretching studies evaluate tendon tissue properties following acute bouts of exercise. It is not the aim of this review to evaluate protocols of such nature, also considering the enormous heterogeneity in the adopted protocols. Regarding longitudinal adaptations, three studies by Konrad and Tilp have applied a 4 × 30 s static stretching protocol [76], a ballistic stretching protocol [77] and a PNF stretching protocol [78] to the plantar flexors until a point of discomfort for six weeks and determined that despite changes in ROM being present, no significant variation in tendon stiffness was present for the passive and ballistic stretching, while a significant decrease for both active and passive stiffness was observed for the PNF protocol. Differences can be observed related to protocols and stretching typology.

Longitudinal interventions taking into account tissue stiffness following stretching in physiology or pathology are warranted since no conclusive evidence can be drawn from the currently included studies, which were all performed by the same research group.

### Muscle Tendon Unit

As discussed in the Muscular Tissue section above, different assessment techniques were adopted to evaluate MTU properties, which may reflect different intrinsic characteristics of specific tissues. In order to include information regarding such specific anatomical areas, all studies regarding MTU have been discussed below.

#### Resistance Training

Only three studies [79–81] evaluated the effects of eccentric RT on the MTU properties. In the first study which involved healthy active subjects [79], the training was carried out daily for six weeks by applying eccentric contractions on the plantar flexor muscles. Each subject had to perform three sets of fifteen repetitions each. At the end of the intervention, stiffness was measured through a dynamometer (passive resistive torque), in combination with ultrasonography. No significant effect on the MTU was found either regarding time or regarding the effect of the intervention. The second study was conducted on eleven untrained men performing 200 maximal eccentric contractions of the quadriceps. The authors observed a decrease in MTU stiffness of 11% following the repeated bout intervention [80]. Measures of passive stiffness were determined from the slope of the curve derived from the passive torque-to-angle relationship. Lastly, the investigation of Fouré et al. [81] evaluated the stiffness of the gastrocnemius muscle–tendon complex following 14 weeks of training in 24 active males. Stiffness was determined as the ratio of changes in external torque and ankle angle between the beginning of the stretch and 60 ms after the stretch. A decreased stiffness was found in the active part of the MTU, while an increase was revealed in its passive components.

No evidence was retrieved regarding adaptations of the MTU to RT performed with concentric contractions or other specific means of delivery. No clear effect regarding MTU stiffness properties following RT performed through eccentric contractions was present.

#### Stretching

Effects of MTU stiffness properties have also been described regarding stretching protocols. All the investigations analysed adaptations of muscles of the lower limb, in particular the plantar flexor muscles [51, 82–85] and the hamstrings [86]. The lengths of the interventions were 4 [82, 86], 5 [51], 6 [84] and 12 weeks [83] and 6 months [85]. All the interventions applied static stretching protocols, except for Mahieu et al. [84] who also included a group undergoing a ballistic protocol. The interventions comprised three to five sets of stretching ranging from 20 to 120 s duration each. All the static stretching investigations reported a significant decrease in stiffness of the MTU, while the only intervention applying ballistic stretching did not show any difference. Measures of stiffness were derived through B-mode ultrasonography [82, 83] measuring the displacement of the MTU from a marked reference point, or by measuring passive torque-to-angle relationship [51, 84–86].

The overall results indicate, despite the limited number of manuscripts, that passive stretching decreases the stiffness of the MTU. Overall, the contradictory results pertaining to the muscular tissue suggest that stretching could act on muscle properties mainly at the level of the MTU rather than the muscle fibres.

### Blood Vessels

Studies taking into account stiffness of blood vessels have all been performed on arteries, and therefore, we will consider arterial stiffness and its measures (described in a subsequent paragraph) to determine blood vessel adaptations to exercise.

#### Resistance Training

Extensive literature has analysed the effects of RT on arterial stiffness, producing several systematic reviews and meta-analyses [87–91]. Despite high-quality reports, an overall effect is not present. When considering the effects of RT on arterial stiffness, exercise parameters such as acute or chronic adaptations or exercise intensity or different populations (especially between young and elderly) need to be taken into account. García-Mateo et al. [89] report that protocols of at least four weeks of duration and two days per week frequency do not impair cardiovascular health in terms of altered arterial stiffness; however, increased stiffness is observed after acute bouts of RT. The exercise intensity of the included interventions ranged from 30 to 100%RM with different effects on arterial stiffness. Increased stiffness was associated with heavy-load RT, while decreased stiffness was associated with low-load RT. It should be noted that the analysed population was predominantly young. Similar results were revealed by Figueroa et al. [90] and Li et al. [91] which highlight that low-load RT may have the potential benefit to lower arterial stiffness. In the study of Figueroa et al., the population analysed was predominantly composed of middle age and elderly people, while those of Li et al. were young. Conversely, Miyachi [87] states that following RT, especially if performed with heavy loads, arterial stiffness increases between 11.6 and 14.3%. Another meta-analysis by Ceciliato et al. [88] found overall no effect of RT on arterial stiffness in healthy subjects. However, the authors, in line with previously discussed investigations, state that all the studies which presented increased arterial stiffness were those with the higher exercise intensities (a mean increase of 13% was evinced in those studies with an exercise load greater than 80% of 1RM, a value very similar to that expressed by Miyachi et al.). It is important to note that all the discussed meta-analysis had a very high heterogeneity of included records, and therefore, the results need to be interpreted with caution. Further attention regarding exercise intensity in specific populations needs to be investigated.

However, common points of the analysed records are that high-intensity RT increases arterial stiffness, while low load may potentially decrease it, in both the elderly and young. The study of Beck et al. was the only one to also include pre-hypertensive patients [92] who underwent 8 weeks of RT 3 days per week, performing 2 sets of 8–12 repetitions to volitional fatigue (⁓70–80% of 1RM). Results of this investigation highlight that RT was able to independently reduce arterial stiffness. A similar outcome was achieved by Cahu Rodrigues et al. [93] by using isometric handgrip training in hypertensive subjects after a 12 week, 4 times a week intervention. The handgrips were performed by continuously gripping the device for 2 min at 30% of each participant’s maximum voluntary contraction. These results have been recently confirmed by a meta-analysis by Lopes et al. which had the aim of understanding the effects of exercise on arterial stiffness in patients with hypertension [94]. It was concluded that isometric RT, which was performed with protocols similar to that of Cahu Rodrigues et al. [93], significantly decreased arterial stiffness. Despite the significant conclusion, it is notable that the model only included two studies, which expresses a high heterogeneity (*I*^2^ = 64%).

The effects of RT on arterial stiffness seem to be mainly influenced by exercise intensity, with low loads with the potential effect of decreasing stiffness and high loads with the potential effect of increasing stiffness. Encouraging results in hypertensive patients are observed after RT protocols. 

#### Aerobic Training

Different forms of AT were retrieved. These included walking, jogging, running, dancing and cycling on a cycle ergometer, using a non-weight-bearing air-braked ergometer.

Despite the heterogeneity of exercise interventions, arterial stiffness in all cases decreased as a consequence of exercise. Findings were also confirmed by other systematic reviews and meta-analyses [94, 95].

A walking and brisk running intervention was carried out for 16 weeks in healthy untrained participants [96]. The intervention proposed continuous activities 2–4 days per week, ranging from 60 to 75% of maximal heart rate reserve. A significant decrease in central but not femoral–ankle pulse wave velocity (PWV) was achieved. A similar intervention proposing continuous running for 30 min in sedentary individuals, three times a week for twelve weeks at an intensity of 60 to 70% of heart rate reserve, also achieved a decrease in arterial stiffness after 8 and 12 weeks from the start of the intervention [97].

A cycle ergometer was adopted in two investigations [98, 99] and used to implement a continuous aerobic activity. In one case [99], a modulation of intensity and duration was carried out. The included participants, which were all sedentary elderly people, were divided into four groups, a 15 min low-intensity AT, 30 min low AT, a 15 min moderate-intensity AT and a 30 min moderate-intensity AT group which were compared to a control group. The intervention was carried out for 8 weeks, twice a week. Arterial stiffness (brachial–ankle and heart–brachial PWV) decreased in all AT groups regardless of duration and intensity. The second investigation [98] was carried out for 8 weeks, three times a week pedalling at 60–70% of VO_2_ max for 45 min. As a consequence of the intervention, a decrease in brachial–ankle PWV was observed. The intervention was carried out on middle-aged adults who were classified as having a high or low fitness level according to their peak oxygen uptake. It is to be noted that no differences were observed between the two groups, suggesting the independent effect of the intervention on arterial stiffness.

Two studies compared continuous aerobic exercise to intermittent exercise [100, 101]. The first investigation [100] proposed a continuous 45 min pedalling on a cycle ergometer at 60–70% of VO_2_ Max and a HIIT (high-intensity interval training) protocol also performed on a cycle ergometer consisting of 6–7 sets of 20 s cycling at an intensity of 170% of VO_2_ Max, both applied to untrained healthy young men. For both protocols, a decrease in carotid–femoral PWV was obtained (− 157.7 ± 45.7 and − 115.3 ± 63.4 m/sec, respectively). The second investigation [101] compared a HIIT to a continuous aerobic exercise performed on a non-weight-bearing air-braked ergometer in sedentary older adults. The intervention lasted 8 weeks and was carried out 4 days per week. The continuous protocol consisted of exercising for 47 min at 70% of peak heart rate (HRpeak), while the HIIT protocol consisted of 4 × 4 min at 90% of HRpeak alternated by 3 × 3 min of active recovery at 70% of HRpeak and a 10 min warm-up and 5 min cool-down at 70% of HRpeak. In both groups, a decrease in arterial stiffness was achieved (carotid–femoral PWV).

Another form of AT was provided in the form of dancing [102]. The activities were carried out, in a sample of untrained elderly for six months, twice a week for 30 min to achieve an increase in heart rate between 110 and 120 bpm. Decreased arterial stiffness was again observed.

An observational study evaluating the effects of lifelong exercise frequency (exercisers were defined as subjects who practised periods of aerobic exercise of at least 30 min per session) on arterial stiffness [103] highlights dose-dependent effects. Higher activity engagement was linked to lower central arterial stiffness values. However, no peripheral adaptation was associated with exercise engagement.

Although this review did not evaluate combined forms of exercise, it is of valuable interest to highlight that when combined forms of exercise (AT plus RT) are evaluated, and in particular, regarding the exercise order, a decrease in arterial stiffness is observed only when the aerobic activity follows the RT protocol [104]. Therefore, there may be a favourable order affecting arterial stiffness which should be considered during exercise prescriptions.

Four interventions took into account pathological cohorts (women with stage two hypertension [105], patients with metabolic syndrome and hypertension [106, 107] and patients with diabetes and hypertension [108]). The exercise protocols comprised stair climbing, continuous and interval aerobic activity, structured on treadmills or cycle ergometers, ranging between 8 weeks and 6 months. In all cases, the AT resulted in significant declines in arterial stiffness of the participants. Results were confirmed by the meta-analysis of Lopes et al. [94].

Although it is well known that increased arterial stiffness and pathological conditions such as hypertension or atherosclerosis, which increase arterial stiffness, are independent factors for increased cardiovascular events [109], there is a strong lack of longitudinal exercise interventions in pathological cohorts. Also, the results of the aforementioned meta-analytic synthesis need to be interpreted with caution since only 5 studies were included in the model. In addition, the majority of the published studies associate different factors with arterial stiffness and cardiovascular events. Therefore, it is premature to define conclusive results regarding the effects of aerobic activity on pathological cohorts.

#### Stretching

A growing body of evidence regarding the effects of stretching and vascular health is present. Two recent meta-analyses have investigated the effects of stretching on arterial stiffness [110, 111]. The results of both investigations are based on a partial overlap of the included records; therefore, these present similar outcomes. Both studies report a decrease in arterial stiffness as a consequence of stretching. The study of Kato et al. [111] only included longitudinal interventions, while the study of Thomas et al. [110] also included acute forms of exercise. The authors of this last investigation state that the moderator analysis regarding the length of the intervention did not highlight significant differences, which underpins that acute stretching bouts may also be able to decrease arterial stiffness, both central (carotid–femoral PWV) and systemic (brachial–ankle PWV). Within the screened populations, obese [112] and non-obese [113] postmenopausal women were included. The study of Wong et al. [112] performed 1 set of 18 active and 20 passive stretches, three times a week for 8 weeks for the main muscle groups. Each stretch was held for 30 s, while Boonpim et al. [113] performed 3 sets of 20 s each, for each main muscle group, five days a week for six weeks. Individual results of these investigations highlight in both cases a reduction in arterial stiffness following the stretching intervention. However, the study of Wong et al. [112] observed a decrease in the augmented index but not in the PWV.

No specific investigation exploring the effects of stretching on arterial stiffness in pathological cohorts is, to date, present.

### Peripheral Nerves

Limited evidence exists regarding the effects of exercise on peripheral nerve stiffness [114]. Only studies analysing the effects of stretching on peripheral nerves were retrieved from our literature search.

#### Stretching

To date, only two stretching studies (of which only one longitudinal intervention) have been performed, both from the same research group, both concluding that a decrease is observed as a consequence of the stretching interventions. Both studies have applied static stretching to the gastrocnemius and the biceps femoris to target the sciatic and tibial nerves. Tissue stiffness was measured in both cases through SWE. The first study [115] provided an acute session of passive stretching revealing a decrease of 13.3% after the stretch. No change was observed in the control group. It is important to note that tissue stiffness was also measured in the targeted stretched muscles (gastrocnemius and the biceps femoris) and none exhibited a significant variation as a consequence of the stretch. The second study [50] applied passive stretching for 12 weeks primarily targeting the plantar flexor muscles. Two stretching modalities were applied, the first with a nerve-directed intention and the second with a muscle-directed intention. Only the nerve-directed intention was able to reduce nerve stiffness, with a mean decrease of 19 and 26% for the sciatic nerve (in its proximal and distal components, respectively), and a mean decrease of 13.7% for the tibial nerve. The muscle-directed stretching reduced muscle stiffness. The results of this study are discussed in the Stretching subsection of the Muscular Tissue section above.

It is still premature to infer that exercise may be able to chronically modulate peripheral nerve stiffness; however, mechanical elongation seemingly has some effects. Future studies should consider these less investigated aspects since nerve stiffness increases either following incorrect postures [116] or in more severe pathological conditions such as carpal tunnel syndrome [117], diabetic peripheral neuropathy [118], rheumatoid arthritis [119] or systemic sclerosis [120].

## Considerations for Pathological Populations

This review has highlighted that exercise may drive specific stiffness adaptations to tissues following a period of intervention. These findings have arisen mainly from healthy but also from clinical populations, despite evidence and intervention studies regarding these latter conditions being limited. However, we believe that further considerations are needed to understand possible tissue adaptations to specific conditions. For this reason, comparison studies between healthy and pathological individuals have also been evaluated and differences caused by specific pathologies arose. For example, in populations affected by musculoskeletal conditions such as low back pain [121–123], neck pain [124] or plantar fasciopathy [125], stiffness of the back and neck muscles and plantar connective tissues resulted higher than matched controls. Increased stiffness in subjects affected by low back pain was not only present in the muscles or connective tissues of the low back but was also observed in measures pertaining to the sciatic nerve [126]. Interestingly, a study by Pamukoff and Blackburn [127] compared stiffness of the plantar flexor muscles of male runners with and without a history of tibial fractures (average time since injury 33 ± 14 months) and noted that those with previous trauma history still presented increased musculoskeletal stiffness.

Other examples can be also applied to vascular and nerve pathology. Patients presenting abdominal aortic aneurysm [128], heart failure [129] and hypertension [130] had significantly higher arterial stiffness compared to healthy individuals. Similarly, also the stiffness of the median nerve resulted increased in patients affected by carpal tunnel syndrome [117], systemic sclerosis [120] and rheumatoid arthritis [119].

Tissue stiffness adaptions have been also observed in other functional pathologies such as migraine [131] or urinary incontinence [132], where increased stiffness of the neck and decreased stiffness of pelvic muscles were observed.

In all above-listed pathological conditions, except for urinary incontinency, stiffness of the different evaluated tissues increased. It is possible that increased stiffness represents a homeostatic mechanism, mediated by inflammatory processes which increase the production by fibroblasts of extracellular matrix components [9], with the function of limiting the movement of specific structures, to reduce the possibility of pain onset or other dysfunctional conditions. However, as known, long-term tissue structural changes can lead to disease progression and development [7]. Specific exercise prescriptions could be implemented to mitigate such phenomena.

## Limitations and Methodological Considerations

This review aimed to identify the effects of exercise on tissue stiffness. We need to highlight that the term stiffness was adapted to fit the needs of each tissue since the assessment methods and the outcomes measured may have differed significantly. A common method adopted by studies pertaining to muscular or connective tissue to determine stiffness was indirectly through a calculation fitted within regression equations of the gradient of force and elongation of muscle fibres during a given task performed through isokinetic dynamometers (usually as the increase in elongation from 50% to 90–100% of a maximal voluntary contraction). Through this, a differentiation between active and passive stiffness in some cases was also calculated, considering the difference between active and passive forces produced by a specific joint from a neutral to a different position and the related tendon or muscle length, and joint angle changes. Isokinetic dynamometry is considered the gold standard for joint stiffness assessment [133]. Limitations of this procedure may be represented by participants body position, muscle fatigue, differences in the angular velocities tested, eccentric or concentric assessments or single or multiple task assessments. However, when standardized procedures and reliable instruments are adopted, the procedures provide valid and reliable measures [134]. Other common methods employed not only for muscular tissue but also for nerve and connective tissue are B-mode ultrasonography and ultrasound elastography, from which shear modulus (the ratio of shear stress to shear strain) or Young’s modulus (the ratio of tensile stress to tensile strain) can be derived. Another frequent method employed was the use of myometers which mechanically determine parameters of stiffness through tissue percussion. Although these non-invasive techniques are able to assess tissue stiffness, limitations to these methods are the lack of standardized protocols, intra- and inter-operator differences during the assessment procedures and the common but fallacious consideration that muscles and tendons are homogeneous entities; therefore, differences in assessment locations may provide heterogeneous data [135].

Other methods are employed to assess arterial stiffness. The most commonly employed is tonometry, which evaluates the time it takes for a pulse wave to travel between two points. These are usually the carotid and the femoral arteries, or the brachial and ankle arteries, to determine central and systemic stiffness, respectively. Knowing the distance between the two points, velocity is calculated, and based on tissue stiffness, the pulse wave velocity may increase (stiffer arteries) or decrease (softer arteries). Derivate measures, as those above described for the muscular tissue, can be calculated for arterial stiffness evaluation. An example is the *β*-stiffness index, which can be measured through carotid ultrasound, which is used to detect the internal luminal diameter during end diastole and end systole of the carotid artery. A ratio is then performed between the ratio of systolic and diastolic pressure, multiplied by its natural logarithm and also by the difference between end systole and end diastole. The obtained number is finally multiplied by the end-diastole diameter [136]. Another derived measure is the augmentation index, which is defined as the ratio of the height of the late systolic shoulder/peak to that of the early systolic shoulder/peak of each pulse [137].

The differences between the assessment methods, as well as the heterogeneity for each exercise protocol within each form of exercise (i.e. (1) resistance training performed through concentric, eccentric or isometric contractions with high or low loads, (2) static, PNF or ballistic stretching and the velocity the stretches are applied, (3) continuous or intermittent aerobic activity) or differences between the analysed populations, prevent defining a dose–response adaptation or in some cases (i.e. stretching for connective tissues) the effect of the exercise interventions.

Longitudinal studies taking into account measures of stiffness as the result of an exercise regime are also very limited.

## Conclusions

Specific tissue adaptations related to each exercise modality seem to be present. Resistance training increases tissue stiffness of tendons and depending on exercise intensity (high or low loads) may also increase or decrease arterial stiffness. However, the effects of resistance training on muscles and muscle–tendon units provide conflicting results related to different exercise modalities.

Plyometric training was seen to increase the stiffness of the muscular tissue; however, no conclusive evidence could be drawn for tendons. No evidence regarding other tissues is to date present.

Aerobic activity was investigated pertaining only to arterial stiffness, which decreases following the exercise protocols.

Passive stretching decreases muscle tendon unit, vessel and nerve stiffness, while unclear effects are seen for muscles and tendons. The effects which were discussed are specific for healthy people.

When analysing pathological populations, it seems that all forms of exercise drive adaptations towards a physiological stiffness state of the targeted tissues. Of interest, resistance training and aerobic training were able to decrease arterial stiffness in hypertensive patients. However, very limited evidence is currently available. Studies evaluating tissue stiffness adaptations using standardized assessment procedures, following specific exercise modalities, should be carried out. Further attention should be paid to peripheral nerves and pathological conditions.

## Data Availability

Data sharing is not applicable to this article as no datasets were generated or analysed during the current study.

## Abbreviations

1RM: 1 Repetition maximum
ACE: Angiotensin-converting enzyme
AT: Aerobic training
HIIT: High-intensity interval training
HRpeak: Peak heart rate
MTU: Muscle tendon unit
PNF: Proprioceptive neuromuscular facilitation
PWV: Pulse wave velocity
ROM: Range of movement
RT: Resistance training
SW: Shear wave
SWE: Shear wave elastography
